# Frequentist Identification of Effective Baskets via the Generalized Information Criteria in Oncology Phase 2 Trials

**DOI:** 10.1002/sim.70673

**Published:** 2026-07-14

**Authors:** Shunya Tanaka, Ryota Ohara, Satoshi Hattori

**Affiliations:** ^1^ Department of Biomedical Statistics Graduate School of Medicine, The University of Osaka Osaka Japan; ^2^ Division of Materials and Manufacturing Science Graduate School of Engineering, The University of Osaka Osaka Japan; ^3^ Integrated Frontier Research for Medical Science Division, Institute for Open and Transdisciplinary Research Initiatives (OTRI) The University of Osaka Osaka Japan

**Keywords:** dual consistency, generalized information criterion, one‐sample Mantel‐Haenszel estimator

## Abstract

Many molecular‐targeted oncology drugs have been successfully developed. The mechanism to target some specific molecules gives us the expectation that the molecular‐target drug is effective over multiple tumor types and histologies. Then, simultaneous evaluation of multiple subtypes is motivated, and the basket trials aim to realize it, in which each subtype is called a basket. Although the single‐arm design with simple exact binomial inference is routinely used for Phase 2 trials in standard oncology drug development, almost all recent proposals of statistical methods for basket trials are Bayesian methods of complexity. To fill the gap, the one‐sample Mantel‐Haenszel procedure was developed, which consists of the exact test of the global null hypothesis, the Mantel‐Haenszel‐type estimation of the treatment effect, and identification of effective baskets via the generalized information criterion (GIC). This paper points out an undesirable feature of the GIC in the previous research and develops an alternative one. The new GIC is free of the undesirable feature and more efficiently borrows information across baskets, which is a relevant feature for basket trials. Through numerical studies, we demonstrate that the new GIC outperforms the original one.

## 1 Introduction

The traditional Phase 2 oncology trials usually focus on a single cancer type or histology and are designed as a single‐arm trial with exact binomial testing or its multistage versions, such as Simon's optimal two‐stage design. This traditional design for Phase 2 studies has been developed in the era of cytotoxic chemotherapies and is still useful today to screen effective molecular‐targeted compounds suitable to investigate their efficacy and safety in large‐scale Phase 3 studies. On the other hand, some molecular‐targeted chemotherapies are expected to be effective over several cancer types or histologies sharing the same target molecules or signal pathways. It motivates us to conduct a Phase 2 study for multiple cancer subtypes or histologies simultaneously instead of focusing on a single one. The basket trial design is to this end, and the subtypes to enroll are called baskets [1, 2, 3].

Since the chemotherapy under investigation is expected to act effectively on multiple baskets through the common mechanism, one may hope to evaluate the efficacy of the chemotherapy efficiently by borrowing information across baskets. By borrowing information, a more precise estimation of response rates and a gain in statistical power can be obtained. In practice, the number of patients of some specific cancer types or histologies can be too small to conduct single‐arm trials separately with sufficient statistical power. If this is the case, borrowing information may make it feasible to evaluate efficacy and safety for such subtypes of a limited number of patients. Thus, in considering statistical methods for basket trials, it is a very important aspect of how one can borrow information across baskets and identify effective baskets that the chemotherapy acts on. Bayesian approach is advantageous at this point. Indeed, recently, many Bayesian methods have been developed [4, 5, 6, 7, 8, 9, 10, 11, 12, 13, 14] and actually applied in clinical research [15, 16, 17]. Correspondingly, a recent review paper [18] on basket trials devotes most space to describing Bayesian methods.

Bakset trials have multiple purposes; one may hope to prove the experimental treatment is certainly effective in a statistically sound manner and discriminate effective baskets to the treatment from ineffective ones. In other words, there are both confirmatory and exploratory aspects. These multiple objectives must be addressed under the severe restriction of limited basket‐specific sample sizes. Bayesian approaches seem to put more emphasis on the exploratory aspect. Indeed, the Bayesian approach is a natural approach to make inference by borrowing external information. On the other hand, most Bayesian methods for basket trials are complicated, relying on strong model assumptions, and then less confirmatory. We believe that even early Phase 2 studies should be designed and analyzed as confirmatorily as possible. Recall that the standard frequentist single‐arm design is based on a simple exact binomial inference for a prespecified testing hypothesis problem and can be computationally easily implemented. Note that the basket trial design can be regarded as a stratified version of the standard single‐arm design. As argued, the majority of recent methodological development is based on the Bayesian approach. Thus, once stratified, the statistical analysis for early Phase 2 oncology clinical trials could be much more complicated. In our view, there is a certain gap between the standard single‐arm design and the current methodology for the basket trials, and it is desirable to develop simple frequentist methods, which are as confirmatorily as possible, to fill the gap. Limited ideas have been introduced for frequentist approaches to basket trials. LeBlanc et al. [19], Cunanan et al. [20] and Krajewska and Rauch [21] proposed methods classified into the “pooling or nothing” approach [18], in which it is determined at interim analyses whether or not the data across baskets are pooled to improve power. Recently, a frequentist model selection approach was proposed with an adaptive lasso approach [22]. Hattori and Morita [23] proposed a frequentist framework called the one‐sample Mantel‐Haenszel (MH) procedure. The idea of the procedure is to borrow information across baskets by estimating the common treatment effect of the risk difference (RD) or the risk ratio (RR). This idea to borrow information by assuming and estimating the common treatment contrast across strata is widely accepted in epidemiology and randomized clinical trials; the MH methods are often used for stratified 2×2 contingency tables [24, 25, 26, 27]. The one‐sample MH procedure for basket trials consists of three steps; (i) testing the global null hypothesis over all the baskets with the exact test by London and Chang [28], (ii) summarizing the global treatment effect over baskets with the MH‐type estimator and (iii) identifying baskets, on which the chemotherapy effectively act, with their proposed generalized information criteria (GIC). All three steps in the one‐sample MH procedure can be implemented with statistics of closed‐form expressions without any complicated iterative computation. The step (i) requires minimal assumptions. The step (ii) relies on the common treatment contrast assumption. But the estimator converges to an interpretable quantity even if the assumption is violated. Thus, the steps (i) and (ii) can be implemented in a confirmatory manner; the test is always valid, and the MH‐type estimator is always interpretable. Ideally, cancer types and subtypes (baskets) should be carefully selected at the design stage with information from basic science and clinical research of similar compounds, and then the effectiveness of the experimental treatments should be proven with prespecified statistical methods of minimal assumptions. The one‐sample MH procedure would contribute to this point in its steps (i) and (ii). On the other hand, it is very difficult in reality to prespecify only effective baskets at the design stage. The step (iii) can attach insights on basket‐specific effects. It has more exploratory aspects, but is still computationally very simple. We would like to put more emphasis on the confirmatory aspect of the basket trial. See Hattori and Morita [23] and its brief summary in Section 2 of the present paper.

In this paper, we focus on step (iii) of the one‐sample MH procedure. The MH estimator in the one‐sample MH procedure was justified under asymptotic considerations. Hattori and Morita [23] showed the dual consistency of the MH‐type estimators and their variance estimators. Dual‐consistency refers to consistency under two frameworks of asymptotic justification [24, 25, 26]; the large‐strata limiting model (Asymptotic 1) and the sparse‐data limiting model (Asymptotic 2). Asymptotic 1 is the framework under which the number of strata (baskets) is fixed, and the number of subjects in each basket goes to infinity, and Asymptotic 2 is that in which the number of subjects in each basket is bounded and the number of baskets goes to infinity.

The generalized information criterion (GIC) [29] is an extension of the Akaike Information Criterion (AIC) [30]. Whereas the AIC was designed for the maximum likelihood estimator, the GIC allows us to use a wide class of estimators represented by a statistical functional. It covers M‐estimators, which are defined as solutions to an estimating equation. With an interpretation of the MH‐type estimators as a solution to an estimating equation or an M‐estimator, applying the general theory of the GIC [29], Hattori and Morita [23] derived the GIC for the one‐sample Mantel‐Haenszel estimators. It implies that the GIC by Hattori and Morita [23] was justified under Asymptotic 2. Then, we call it the $GIC_{2}$ in this paper. Hattori and Morita [23] proposed clustering baskets with the $GIC_{2}$ into some sets of baskets, within each of which the common parameter (RD or RR) assumption holds and then identify effective baskets according to the resulting subclassification of baskets. We point a pathological issue of the $GIC_{2}$ for practical use out. The GIC has a penalty term, which comes from a bias correction of the Kullback‐Leibler divergence. It plays a role to avoid selecting a too complicated model overly fitting to the data. We found that the penalty term of the $GIC_{2}$ may not work well and propose an alternative GIC. We show that the MH‐type estimators are regarded as a solution to an alternative estimating equation, which is justified under Asymptotic 1. According to this interpretation, we derived the GIC under Asymptotic 1, called the $GIC_{1}$. It has a different penalty term from the $GIC_{2}$ and is free of the pathological nature of the $GIC_{2}$. Simulation studies and analyses of some real datasets indicated that the $GIC_{1}$ borrowed more information across baskets appropriately and outperformed the $GIC_{2}$ under almost all the scenarios in the simulation study.

The organization of the rest of the paper is as follows. In Section 2, to make the paper self‐contained, a brief review of the motivation and the outline of the one‐sample MH procedure by Hattori and Morita [23] are given. Section 3 is the main contribution of the paper. We improve the step (iii) of the one‐sample MH procedure for the identification of effective baskets. We begin by reviewing the $GIC_{2}$ and presenting a representation and an interpretation of the penalty term of the $GIC_{2}$. Then, the pathological nature of the $GIC_{2}$ is pointed out, and an alternative one, called the $GIC_{1}$, is proposed. The performance of the $GIC_{1}$ and $GIC_{2}$ are investigated through analyzing real datasets and simulation studies in Sections 4 and 5, respectively. We conclude the paper with some discussions in Section 6.

## 2 Preliminary

Let us consider a single‐arm oncology basket trial of K baskets with a binary tumor response as the primary endpoint. For the kth basket (k=1,2,…,K), let $n_{k}$ and $Y_{k}$ be the number of subjects enrolled and that responded to the treatment in the kth basket. We assume that conditional on $n_{k}$, $Y_{k}$ follows the binomial distribution $Bin(n_{k},\pi_{k})$, where $\pi_{k}$ is the true response rate of the kth basket. Consider the following hypothesis testing problem; the null hypothesis is

(1) $$\begin{array}{l} H_{0}:\pi_{k}=\pi_{k,0} \end{array}$$

for any k, where $\pi_{k,0}$ is a basket‐specific clinically meaningful minimum overall response rate and should be set accounting for historical data and clinical perspective. Hattori and Morita [23] proposed a frequentist framework for the statistical analysis of basket trials, called the one‐sample MH procedure. It consists of the following three steps;

i. The primary analysis is testing the global null hypothesis (1) with the exact conditional test by London and Chang [28].
ii. The treatment effect is summarized as the common risk difference (RD) or the risk ratio (RR) relative to $\left\{\pi_{k,0}\right\}$ with the MH‐type estimator.
iii. The subclasses of effective baskets are identified using the GIC.

In the step (i), the test statistic $T_{w}=\sum_{k=1}^{K}w_{k}Y_{k}$ is used, where $w_{k}$ is the weight for the kth basket. Hattori and Morita [23] proposed to use the constant weight $w_{k}=1$ over the basket when the RD was used in step (ii) and the weight $w_{k}=\pi_{k,0}^{-1}$ when the RR was used. Hattori and Morita [23] proposed the one‐sample Mantel‐Haenszel risk‐difference (MH‐RD) and risk‐ratio (MH‐RR) estimators for the purpose of step (ii).

The MH‐RD estimator is defined as

(2) $$\begin{array}{ll} {\hat{\Delta }}^{RD} & =\frac{\sum_{k=1}^{K}(Y_{k}-n_{k}\pi_{k,0})}{\sum_{k=1}^{K}n_{k}}=\frac{\sum_{k=1}^{K}R_{k}^{RD}}{\sum_{k=1}^{K}S_{k}^{RD}}, \end{array}$$

where $R_{k}^{RD}=Y_{k}-n_{k}\pi_{k,0}$ and $S_{k}^{RD}=n_{k}$. The common RD assumption is defined as follows;

(3) $$\begin{array}{l} \Delta^{RD}=\Delta_{k}^{RD}=\pi_{k}-\pi_{k,0}. \end{array}$$

for k=1,2,…,K. Hattori and Morita [23] showed that if the common RD assumption (3) holds, the MH‐RD estimator is consistent to the common RD. The consistency was established under the two asymptotic models (dual consistency);

Asymptotic 1 (the large strata limiting model): K is fixed and $n_{k}\to \infty$ and $n_{k}/n\to q_{k}\in (0,1)$,

and

Asymptotic 2 (the sparse data limiting model): K→∞ and $n_{k}$ is fixed and bounded over k.

The dual consistency is a desirable property for sparse data arising in basket trials. In clinical trials, it is desirable that the statistical method for the primary objective is prespecified. In basket trials, ideally, only baskets expected to be effective should be included and then the one‐sample MH‐RD procedure borrows information efficiently across baskets if the RDs are common over baskets. In reality, the common RD assumption (3) may not hold, and similar improvement over $\pi_{k,0}$ may not exist for some baskets. Hattori and Morita [23] showed that the MH‐RD estimator (2) converges in probability to $\sum_{k=1}^{K}q_{k}\Delta_{k}^{RD}$ even if the common RD assumption is violated under Asymptotic 1 (see Section 4.3 of Hattori and Morita (2023)). Recall that $q_{k}$ is the limit of $n_{k}/n$, and then it can be interpreted as the average of the basket‐specific RDs weighted by sample size. This interpretation also holds under Asymptotic 2. Coupled with the validity of the conditional test, the steps (i) and (ii) can be conducted in a confirmatory manner; even if the common RD assumption is violated, the prespecified conditional test and the MH‐RD estimator are valid and interpretable.

Hattori and Morita [23] also considered inference under the common RR assumption; for k=1,2,…,K,

(4) $$\begin{array}{l} \Delta^{RR}=\Delta_{k}^{RR}=\frac{\pi_{k}}{\pi_{k,0}}. \end{array}$$

The one‐sample MH‐RR estimator was defined as

(5) $$\begin{array}{ll} {\hat{\Delta }}^{RR} & =\frac{\sum_{k=1}^{K}w_{k}Y_{k}}{\sum_{k=1}^{K}w_{k}n_{k}\pi_{k,0}}=\frac{\sum_{k=1}^{K}R_{k}^{RR}}{\sum_{k=1}^{K}S_{k}^{RR}} \end{array}$$

where $w_{k}$ is a prespecified weight for the kth basket, $R_{k}^{RR}=w_{k}Y_{k}$ and $S_{k}^{RR}=w_{k}n_{k}\pi_{k,0}$. If the common RR assumption (4) holds, the MH‐RR estimator (5) converges in probability to the common RR. Hattori and Morita [23] recommended to set the weight in the definition of the estimator (5) as $w_{k}=\pi_{k,0}^{-1}$ since the MH‐RR estimator with $w_{k}=\pi_{k,0}^{-1}$ converges in probability to $\sum_{k=1}^{K}q_{k}\Delta_{k}^{RR}$ and then has an interpretation as the average of the basket‐specific RRs weighted by sample size even if the common RR assumption does not hold. We employ this weight and call the resulting MH‐RR estimator the MH‐iwRR estimator.

Although both the MH‐RD and MH‐iwRR estimators preserve certain interpretability under violation of the common parameter assumption, interpretation as the weighted average is not sufficiently appealing, and one would like to identify baskets for which the treatment is effective. Hattori and Morita [23] proposed the GIC for this purpose in step (iii). It is the primary focus of this paper, and is discussed in the next section comprehensively.

## 3 Generalized Information Criteria for Effective Basket Identification

### 3.1 The Criterion Under Asymptotic 2 (The Sparse Data Limiting Model)

As done in Hattori and Morita [23], we treat the MH‐RD and the MH‐RR estimators in a unified way. Let Δ be $\Delta^{RD}$ and $\Delta^{RR}$ when the RD and the RR are considered, respectively, and $\hat{\Delta }$ is defined in a similar way. Similarly, $R_{k}$ and $S_{k}$ are defined. Define $h_{k}(x)=\pi_{k,0}+x$ for the MH‐RD and $h_{k}(x)=\pi_{k,0}x$ for the MH‐RR. Then, $\pi_{k}=h_{k}(\Delta )$ holds for both the MH‐RD and the MH‐RR. One can see that $\hat{\Delta }$ is regarded as the solution [23] to

(6) $$\begin{array}{l} U(\Delta )=\overset{K}{\underset{k=1}{\sum }}(R_{k}-\Delta S_{k})=0. \end{array}$$

The GIC proposed by Konishi and Kitagawa [29] is an extension of the AIC [30] to allow estimators other than the maximum likelihood estimator. The GIC covers M‐estimators, that is, the estimators defined by a solution to an estimating equation. It is summarized as a lemma in Appendix A.

Applying the general theory of the GIC for M‐estimators (see Theorem 3.1 of Konishi and Kitagawa (1996) and Appendix A of the present paper) to the estimating equation (6), Hattori and Morita [23] proposed the GIC,

(7) $$\begin{array}{l} GIC_{2}=-\overset{K}{\underset{k=1}{\sum }}\left[Y_{k}logh_{k}(\hat{\Delta })+(n_{k}-Y_{k})log(1-h_{k}(\hat{\Delta }))\right]+bias_{2}(\hat{\Delta }), \end{array}$$

where

(8) $$\begin{array}{ll} bias_{2}(\Delta ) & ={\left(\overset{K}{\underset{k=1}{\sum }}S_{k}\right)}^{-1}\overset{K}{\underset{k=1}{\sum }}(R_{k}-\Delta S_{k})\left\{Y_{k}\frac{{\dot{h}}_{k}(\Delta )}{h_{k}(\Delta )}-(n_{k}-Y_{k})\frac{{\dot{h}}_{k}(\Delta )}{1-h_{k}(\Delta )}\right\} \end{array}$$

and ${\dot{h}}_{k}(x)=dh_{k}(x)/dx$ (see the Equation (10) of Hattori and Morita [23] and Appendix B of the present paper). Note that the general theory of the GIC by Konishi and Kitagawa [29] relied on the asymptotic theory and the GIC in (7) was justified under the Asymptotic 2 since the asymptotic property of the MH estimator was determined by the behavior of the estimating equation (6) as K went to infinity. Then, we refer to the GIC in (7) as the $GIC_{2}$ since we will discuss an alternative one, called the $GIC_{1}$.

The first term of the $GIC_{2}$ in (7) is the minus log‐likelihood, which measures the goodness‐of‐fit of the model, and the bias (8) is regarded as the complexity of the model and acts as a penalty to overfitting of the model to data. The bias term of the information criteria is derived as an estimate of the expected bias of the Kullback‐Leibler divergence, which measures the distance between the fitted model and the true one. The GIC was derived without assuming the evaluated models were correctly specified. On the other hand, the AIC was under the assumption that the model was correctly specified [29]. From the general theory by Konishi and Kitagawa [29], if the fitted model is correct, the bias term (8) reduces to the number of unknown parameters to estimate (see Theorem 3.1 of Konishi and Kitagawa [29]). In other words, the GIC reduces to AIC=−loglikelihood+p, where p is the number of parameters to estimate. If the common RD assumption holds, we have a single unknown parameter of the common RD and then p=1. Hattori and Morita [23] proposed to classify the baskets or to select the model minimizing the $GIC_{2}$ among the candidate models. Here, the model implies a partitioning of the baskets. For simplicity of explanation, let us consider a situation of K=4 and the set of the indexes of the baskets denoted by {1,2,3,4}. We consider a partitioning of the indexes as a model, within each element of which the common parameter assumption holds. For example, if we assume the common parameter assumption holds over all four baskets, we denote the model by [1,2,3,4]. If we assume that the common parameter assumption holds between the baskets 1 and 2 and does between the baskets 3 and 4, the model corresponds to the partitioning {1,2}⋃{3,4} and is denoted by [1,2/3,4]. Let the common parameter (RD or RR) be denoted by $\Delta_{1,2,3,4}$ and its MH‐RD or MH‐iwRR estimator by ${\hat{\Delta }}_{1,2,3,4}$ for the model [1,2,3,4]. The $GIC_{2}$ for the model is denoted by the $GIC_{2}^{\left[1,2,3,4\right]}$ and is given by

(9) $$\begin{array}{ll} GIC_{2}^{\left[1,2,3,4\right]} & =-\overset{4}{\underset{k=1}{\sum }}\left[Y_{k}logh_{k}({\hat{\Delta }}_{1,2,3,4})\right.\\ & \;\left.+(n_{k}-Y_{k})log(1-h_{k}({\hat{\Delta }}_{1,2,3,4}))\right]+bias_{2}({\hat{\Delta }}_{1,2,3,4}). \end{array}$$

For the model [1,2/3,4], the common parameters for {1,2} and {3,4} are denoted by $\Delta_{1,2}$ and $\Delta_{3,4}$, and their MH‐RD or MH‐iwRR estimators are denoted by ${\hat{\Delta }}_{1,2}$ and ${\hat{\Delta }}_{3,4}$, respectively. Similar notation is used for other models. The $GIC_{2}$ for the model [1,2/3,4] is calculated by $GIC_{2}^{\left[1,2/3,4\right]}=GIC_{2}^{\left[1,2\right]}+GIC_{2}^{\left[3,4\right]}$, each term of which is defined in a similar way to (9). For the model [1,2,3,4], the only parameter is $\Delta_{1,2,3,4}$, and then the number of the parameter to estimate is one. The model [1,2/3,4] has two parameters, $\Delta_{1,2}$ and $\Delta_{3,4}$. The model [1,2/3,4] is more flexible and is likely to fit better to data. The bias term acts as a penalty to separate baskets into heterogeneous subclasses, and the $GIC_{2}$ is expected to cluster baskets into some subclasses within which the treatment effects are homogeneous and among which are not.

### 3.2 An Interpretation of the Bias Term (8) and a Pathological Nature of the $GIC_{2}$

We present an interpretation of the bias term of the $GIC_{2}$, which was not addressed in Hattori and Morita [23]. It is represented as

(10) $$\begin{array}{l} bias_{2}(\hat{\Delta })={\left(\overset{K}{\underset{k=1}{\sum }}S_{k}\right)}^{-1}\overset{K}{\underset{k=1}{\sum }}n_{k}\frac{{\left\{Y_{k}-n_{k}h_{k}(\hat{\Delta })\right\}}^{2}}{n_{k}h_{k}(\hat{\Delta })(1-h_{k}(\hat{\Delta }))} \end{array}$$

It is related to the test statistic for the chi‐squared goodness‐of‐fit test; the denominator of the summand $n_{k}h_{k}(\hat{\Delta })(1-h_{k}(\hat{\Delta }))$ is approximated by $n_{k}h_{k}(\hat{\Delta })$, and then, one can see that $\frac{{\left\{Y_{k}-n_{k}h_{k}(\hat{\Delta })\right\}}^{2}}{n_{k}h_{k}(\hat{\Delta })(1-h_{k}(\hat{\Delta }))}$ is approximately the test statistics for the chi‐square goodness‐of‐fit test, $\frac{{\left\{Y_{k}-n_{k}h_{k}(\hat{\Delta })\right\}}^{2}}{n_{k}h_{k}(\hat{\Delta })}$. Thus, if the common parameter assumption is violated, it is likely to take a larger value and then put a larger penalty on the information criterion. With an overly simplified model of poor fit, the first term of the $GIC_{2}$ (minus log‐likelihood) gets larger. At the same time, since the common parameter assumption does not hold, the goodness‐of‐fit statistics gets larger and then the bias term puts a larger penalty on the model. Thus, the bias term acts as a penalty not only for the complexity of the model but also for the poor fit of the model.

On the other hand, we point a pathological property of the $GIC_{2}$ out. Suppose we consider a model with multiple subclasses of baskets, some of which consist of a single basket. For example, in the case of K=4, we consider a model [1,2,3/4]. We call a single basket like {4} the isolated basket, which does not share information with any other basket. For the isolated basket {4}, the MH‐RD estimator becomes $Y_{4}/n_{4}-\pi_{4,0}$. Then, the bias term is algebraically 0 from the definition of (10). That implies the bias term of the $GIC_{2}$ in (10) does not give any penalty for the part of the isolated baskets. That is, in the presence of such isolated baskets, the bias term does not work as a penalty for the complexity of the model. For the model of the most precise separation [1/2/3/4], the MH‐RD estimator for each basket is the maximum likelihood estimator, which minimizes the first term of the $GIC_{2}$. In theory, as outline in Section 2, the penalty term agrees with the number of baskets and acts as the penalty to select too precise separation. However, with the pathological nature of the $GIC_{2}$, the bias term of the $GIC_{2}$ becomes zero and then no penalty is imposed. Then, the model [1/2/3/4], which consists of only isolated baskets, has the minimum $GIC_{2}$ even if the true structure can be described with a simpler model and then some baskets can be combined.

### 3.3 An Alternative GIC: $GIC_{1}$

Let $n=\sum_{k=1}^{K}n_{k}$ be the total number of subjects. For i=1,2,⋯,n, denote the binary response of the ith subject by $Y^{\left(i\right)}$. Let $D_{k}^{\left(i\right)}$ be an indicator function for the membership of the kth basket; if the subject i belongs to the kth basket, $D_{k}^{\left(i\right)}=1$ and =0 otherwise. Then, $n_{k}=\sum_{i=1}^{n}D_{k}^{\left(i\right)}$ and $Y_{k}=\sum_{i=1}^{n}D_{k}^{\left(i\right)}Y^{\left(i\right)}$. Let us begin with the MH‐RD estimator. Note that $R_{k}^{RD}=Y_{k}-n_{k}\pi_{k,0}=\sum_{i=1}^{n}D_{k}^{\left(i\right)}\{Y^{\left(i\right)}-\pi_{k,0}\}$ and $S_{k}^{RD}=n_{k}=\sum_{i=1}^{n}D_{k}^{\left(i\right)}$. Then, the estimating equation (6) is represented by

(11) $$\begin{array}{l} U^{RD}(\Delta^{RD})=\overset{n}{\underset{i=1}{\sum }}\overset{K}{\underset{k=1}{\sum }}D_{k}^{\left(i\right)}\{Y^{\left(i\right)}-(\pi_{k,0}+\Delta^{RD})\}=0 \end{array}$$

For the case of the MH‐iwRR estimator, $R_{k}^{RR}=w_{k}Y_{k}=\sum_{i=1}^{n}D_{k}^{\left(i\right)}w_{k}Y^{\left(i\right)}$ and $S_{k}^{RR}=\sum_{k=1}^{K}w_{k}n_{k}=\sum_{i=1}^{n}w_{k}D_{k}^{\left(i\right)}$ with $w_{k}=\pi_{k,0}^{-1}$. The estimating equation (6) is represented by

(12) $$\begin{array}{l} U^{iwRR}(\Delta^{iwRR})=\overset{n}{\underset{i=1}{\sum }}\overset{K}{\underset{k=1}{\sum }}w_{k}D_{k}^{\left(i\right)}\{Y^{\left(i\right)}-\pi_{k,0}\Delta^{RR}\}=0 \end{array}$$

Then, the estimating equations (11) and (12) have the following unified representation;

(13) $$\begin{array}{l} U(\Delta )=\overset{n}{\underset{i=1}{\sum }}\overset{K}{\underset{k=1}{\sum }}w_{k}D_{k}^{\left(i\right)}\{Y^{\left(i\right)}-h_{k}(\Delta )\}=0, \end{array}$$

where $w_{k}=1$ and $h_{k}(x)=\pi_{k,0}+x$ for the MH‐RD and $w_{k}=\pi_{k,0}^{-1}$ and $h_{k}(x)=\pi_{k,0}x$ for the MH‐iwRR. It is an alternative representation of (6) as a sum over i and enables us to consider the GIC under Asymptotic 1. It is denoted by the $GIC_{1}$. Applying the general theory of the GIC for the M‐estimator (see Appendix A), the $GIC_{1}$ is given by

$$\begin{array}{l} GIC_{1}=-\overset{K}{\underset{k=1}{\sum }}\left[Y_{k}logh_{k}(\hat{\Delta })+(n_{k}-Y_{k})log(1-h_{k}(\hat{\Delta }))\right]+bias_{1}(\hat{\Delta }) \end{array}$$

The first term of the $GIC_{1}$ is the same as that of the $GIC_{2}$. The bias term is calculated as

(14) $$\begin{array}{l} bias_{1}(\Delta )={\left(\overset{K}{\underset{k=1}{\sum }}S_{k}\right)}^{-1}\overset{K}{\underset{k=1}{\sum }}\left[\frac{\{1-2h_{k}(\Delta )\}Y_{k}+h_{k}{\left(\Delta \right)}^{2}n_{k}}{h_{k}(\Delta )\{1-h_{k}(\Delta )\}}\right] \end{array}$$

A derivation of the $GIC_{1}$ is given in Appendix C. The bias term of the $GIC_{1}$ is not zero for isolated baskets, and then the $GIC_{1}$ does not suffer from the pathological phenomena observed for the $GIC_{2}$.

We propose to select the best model minimizing the $GIC_{1}$ over a class of candidate models. For example, we may set a class of models consisting of all the models of two subclasses and the model assuming the common parameter assumption. We call this approach the two‐subclass approach. Or, we may consider the class of all the subclassifications. We call it the all‐subclass approach.

## 4 Examples

### 4.1 Vemurafenib for BRAF‐Positive Nonmelanoma Cancer

Hyman et al. [15] reported results of a basket trial for vemurafenib in BRAF V600 mutation‐positive nonmelanoma cancers. Six cancer types were predefined as baskets: anaplastic thyroid cancer (ATC), Erdheim‐ Chester disease of Langerhans'‐cell histiocytosis (ECD/LCH), cholangiocarcinoma (CCA), colorectal cancer treated by vemurafenib (CRC‐V), colorectal cancer treated by vemurafenib and cetuximab (CRC‐VC), and non‐small‐cell lung cancer (NSCLC). Simon's two‐stage method [31] was used separately for each basket with the tumor response at week 8 as the primary endpoint. The clinically meaningful minimum response rate was defined as $\pi_{k,0}=0.15$ for all the baskets. Hattori and Morita [23] used this study for illustrating the one‐sample Mantel‐Haenszel procedure. The results of the exact tests, the MH‐RD and MH‐iwRR estimators, as well as the number of subjects $n_{k}$ and that of responders $Y_{k}$ of each basket, were presented in Table 1 of Hattori and Morita [23]. Here, we argue based on the RD and then use the test statistic $T=\sum_{k=1}^{K}Y_{k}$. The one‐sided *p*‐value of the exact test was *p* = 0.0710, which was calculated with 10,000 simulated samples from the null distribution. The MH‐RD estimate was 0.064 (95% confidence interval CI: −0.017, 0.146). From the basket‐specific RD estimates (see Table 1 of Hattori and Morita [23]), heterogeneity of the risk difference was suggested.

**TABLE 1:** Top 5 models with respect to the ${GIC}_{1}$ *and those to the* ${GIC}_{2}$ *for Vemurafenib data*.

Candidate	Rank	${GIC}_{1}$		${GIC}_{2}$	
		GIC	Model	GIC	Model
All‐subclass	1	36.527	[1,2,6/3,4/5]	33.933	[1/2/3/4/5/6]
	2	37.056	[1,2,6/3,4,5]	33.935	[1/2,6/3/4/5]
	3	37.173	[1,2,6/3/4/5]	34.273	[1,2,6/3/4/5]
	4	37.238	[1,3/2,6/4/5]	34.293	[1,6/2/3/4/5]
	5	37.288	[1/2,6/3,4/5]	34.319	[1,2/3/4/5/6]
Two‐subclass	1	37.056	[1,2,6/3,4,5]	35.494	[1,2,6/3,4,5]
	2	37.769	[1,2,3,6/4,5]	36.501	[1,2,3,6/4,5]
	3	38.515	[1,3,4,5/2,6]	37.584	[1,3,4,5/2,6]
	4	39.806	[1,4,5/2,3,6]	39.623	[1,4,5/2,3,6]
	5	40.950	[1,2,5,6/3,4]	40.745	[1,2,5,6/3,4]

We calculated the $GIC_{1}$s and $GIC_{2}$s of all possible models from the two sub‐classifications, as well as those under the common RD assumption. Then, we compared in total 32 models with the GIC (the two‐subclass approach). In Table 1, the 5 top models with respect to the $GIC_{1}$, as well as those with respect to the $GIC_{2}$, are listed. Recall that the $GIC_{2}$ is the criterion discussed in Hattori and Morita [23]. Under the two‐subclass approach, both GICs suggested the same sub‐classification of [1,2,6/3,4,5] and the common RD over the baskets 1,2 and 6 were estimated as 0.250 (95% CI: 0.093,0.407), which successfully excluded the null value of 0 for the RD. We also present the results on the all‐subclass approach; all the sub‐classifications of {1,2,3,4,5,6} are considered as candidate models and select the model minimizing the $GIC_{1}$ or $GIC_{2}$ over the candidates. The $GIC_{1}$ selected the sub‐classification [1,2,6/3,4/5]; a similar but slightly more precise sub‐classification than that under the two‐subclass approach was selected. On the other hand, the sub‐classification of [1/2/3/4/5/6] was selected with the $GIC_{2}$. That is, a pathological phenomenon in Section 3.2 was actually observed; the bias term of the $GIC_{2}$ does not act properly as a penalty term with isolated baskets and indeed, the model with only isolated baskets was selected. The MH‐RD estimates based on the selected model under each approach were presented in Table 2. The $GIC_{1}$ suggested statistically significant treatment effects over the clinically meaningful minimum overall response rate $\pi_{k,0}$ for the baskets 1, 2 and 6. As anticipated, we observed that the $GIC_{2}$ coupled with the all‐subclass approach failed to borrow information across baskets and then resulted in less biased but more variable estimates without borrowing. On the other hand, in this example, with the two‐subclass approach, $GIC_{2}$ selected the same model [1, 2, 6/3, 4, 5] as $GIC_{1}$; the two‐subclass approach would weaken the impact of the pathological nature of the $GIC_{2}$. The $GIC_{1}$ allows us to search the best model over a wider class of models.

**TABLE 2:** Sub‐class specific RD based on the selected models with $GIC_{1}$ *or* $GIC_{2}$ *for Vemurafenib data; NA implies Not Available*.

Candidate	GIC	Model	Baskets	MH‐RD
All‐subclass	$GIC_{1}$	[1,2,6/3,4/5]	1,2,6	0.250 (0.093,0.407)
			3,4	−0.091 (−0.173, −0.010)
			5	−0.150 (NA, NA)
	$GIC_{2}$	[1/2/3/4/5/6]	1	0.136 (−0.226,0.497)
			2	0.279 (0.010,0.548)
			3	−0.025 (−0.270,0.220)
			4	−0.112 (−0.187, −0.036)
			5	−0.150 (NA, NA)
			6	0.271 (0.043,0.499)
Two‐subclass	$GIC_{1}$	[1,2,6/3,4,5]	1,2,6	0.250 (0.093,0.407)
			3,4,5	−0.105 (−0.168, −0.042)
	$GIC_{2}$	[1,2,6/3,4,5]	1,2,6	0.250 (0.093,0.407)
			3,4,5	−0.105 (−0.168, −0.042)

### 4.2 Imatinib for Advanced Sarcoma

Chugh et al. [17] reported results of a basket trial for imatinib in subjects with advanced sarcoma. It had 10 subtypes (baskets) of sarcoma. The primary endpoint was the clinical benefit rate (CBR), which was defined as Complete Response, Partial Response within 16 weeks or lasting Stable Disease at least 16 weeks. A Bayesian hierarchical model (BHM) [12] was predefined for the primary analysis. The clinically meaningful minimum response rate as 0.3 throughout the baskets, and basket‐specific efficacy was adaptively assessed by monitoring $P(\pi_{k}>0.3|data)$ for k=1,2,…,10, borrowing information across baskets. For all the baskets, clinically meaningful efficacy was not established. We use this data for illustration as done by Hattori and Morita [23]. We set the clinically meaningful minimum response rate at $\pi_{k,0}=0.1$ for all the baskets for illustration. In Table 4 of Hattori and Morita [23], the number of subjects $n_{k}$, that of responders $Y_{k}$ and the response rate with Pearson‐Clopper two‐sided 95% confidence intervals are summarized. Here, we focus on the MH‐iwRR estimator. The one‐sided *p*‐value of the exact test was 0.0120 with the test statistic $T_{iw}=\sum_{k=1}^{K}\pi_{k,0}^{-1}Y_{k}$. The MH‐iwRR estimate was 1.564 (1.029, 2.100). The exact test and the Mantel‐Haenszel estimator suggested that Imatinib is effective at least for some subtypes of sarcoma. Hattori and Morita [23] applied the chi‐squared goodness‐of‐fit test, which gave *p* = 0.784 and did not suggest substantial violation of the common RR assumption.

In Table 3, the top five models with respect to the $GIC_{1}$ and those with respect to the $GIC_{2}$ are listed on the all‐subclass and two‐subclass approaches to define the candidate models. With the $GIC_{1}$, the model [1,3,6,10/2,9/4,5,7,8] was selected with the minimum $GIC_{1}$ under the all‐subclass approach. With the $GIC_{2}$, the models [1/2,9/3/4/5/6/7/8/10] and [1/2/3/4/5/6/7/8/9/10] were ranked at the top and second‐best models under the all‐subclass approach. Although, as argued in Section 3.2, the latter model should have the minimum $GIC_{2}$ due to the pathological nature of the $GIC_{2}$, the former model was selected probably with estimated penalty terms. The top two models had very close $GIC_{2}$. From the results with the $GIC_{1}$ and basket‐specific response rates (see Table 3 of Hattori and Morita [23]), subclassification into two or three subclasses seems relevant. In Table 4, the MH‐iwRRs were summarized based on the selected models. As observed in the previous example, the $GIC_{2}$ worked poorly as expected; the $GIC_{2}$ under the all‐subclass approach gave too precise subclassification and then led to less precise estimates of the RR for all the baskets. The $GIC_{1}$ protected a too precise subclassification with proper control of the bias term and then led to statistically significant RR for baskets 4, 5, 7, and 8. The $GIC_{2}$ with the two‐subclass approach selected the best model [1,2,3,6,9,10/4,5,7,8], which is substantially different from that with the all‐subclass approach as given in Table 4. As observed with the first example in Section 4.1, the two‐subclass approach could weaken the influence of the pathological nature of the $GIC_{2}$ and then led to more borrowing across baskets. The $GIC_{1}$ realized it in a more flexible manner.

**TABLE 3:** Top 5 models with the ${GIC}_{1}$ *and those with the* ${GIC}_{2}$ *for Imatinib data*.

Candidate	Rank	$GIC_{1}$		$GIC_{2}$	
		GIC	Model	GIC	Model
All‐subclass	1	74.946	[1,3,6,10/2,9/4,5,7,8]	73.237	[1/2,9/3/4/5/6/7/8/10]
	2	74.946	[1,3,6,10/2/4,5,7,8/9]	73.237	[1/2/3/4/5/6/7/8/9/10]
	3	75.058	[1,3,6,8,10/2,9/4,5,7]	73.243	[1/2,9/3/4/5/6/7,8/10]
	4	75.058	[1,3,6,8,10/2/4,5,7/9]	73.243	[1/2/3/4/5/6/7,8/9/10]
	5	75.093	[1,3,6/2,9/4,5,7,8,10]	73.253	[1/2,9/3/4,8/5/6/7/10]
Two‐subclass	1	75.643	[1,3,4,5,6,7,8,10/2,9]	79.663	[1,2,3,6,9,10/4,5,7,8]
	2	76.018	[1,3,4,5,6,7,8,9,10/2]	79.705	[1,2,3,6,8,9,10/4,5,7]
	3	76.628	[1,4,5,7,8,10/2,3,6,9]	79.871	[1,3,4,5,6,7,8,10/2,9]
	4	76.630	[1,2,3,6,9/4,5,7,8,10]	80.007	[1,2,3,6,10/4,5,7,8,9]
	5	76.697	[1,2,3,6,9,10/4,5,7,8]	80.083	[1,2,3,6,8,10/4,5,7,9]

**TABLE 4:** Sub‐class specific iwRR based on the selected models with ${GIC}_{1}$ *or* ${GIC}_{2}$ *for Imatinib data; NA implies Not Available*.

Candidate	GIC	Model	Baskets	MH‐iwRR
All‐subclass	$GIC_{1}$	[1,3,6,10/2,9/4,5,7,8]	1,3,6,10	1.184 (0.440, 1.928)
			2,9	0.000 (NA, NA)
			4,5,7,8	2.159 (1.280, 3.038)
	$GIC_{2}$	[1/2,9/3/4/5/6/7/8/10]	1	1.333 (−0.447, 3.114)
			2,9	0.000 (NA, NA)
			3	0.833 (−0.800, 2.467)
			4	2.143 (0.595, 3.691)
			5	2.414 (0.829, 3.999)
			6	1.034 (−0.094, 2.163)
			7	1.923 (0.378, 3.468)
			8	2.000 (−1.920, 5.920)
			10	1.500 (−0.106, 3.106)
Two‐subclass	$GIC_{1}$	[1,3,4,5,6,7,8,10/2,9]	1,3,4,5,6,7,8,10	1.707 (1.123, 2.292)
			2,9	0.000 (NA, NA)
	$GIC_{2}$	[1,2,3,6,9,10/4,5,7,8]	1,2,3,6,9,10	0.989 (0.367, 1.611)
			4,5,7,8	2.159 (1.288, 3.038)

## 5 Simulation Study

### 5.1 Data Generation

In this section, we demonstrate results of the simulation study to evaluate the performance of the $GIC_{1}$ in contrast to the $GIC_{2}$. As done in Hattori and Morita [23], we generated simulation datasets following the setting by Zhou and Ji [13], in which several Bayesian methods were compared. Same as Zhou and Ji [13], we considered 7 scenarios. The first and the second scenarios were under the common RD assumption over all the baskets. Scenario 1 was under the global null (GN) hypothesis, in which all the baskets had no treatment effect. Scenario 2 was under the global alternative (GA), in which the treatment was effective over all the baskets. We compare performance to identify the effective baskets with the $GIC_{1}$ and $GIC_{2}$. By referring to the results of the simulation study by Zhou and Ji [13], one may compare them with some representative Bayesian methods. In addition, we present the results with the multi‐exchangeability model (MEM) by Hobbs and Landin [7]. See Section 5.3 for more details. We have K=4 baskets of ($n_{1}$, $n_{2}$, $n_{3}$, $n_{4}$)=(20, 20, 10, 10) and $\pi_{k,0}=0.1$ for k=1,2,…,4. Seven scenarios for ($\pi_{1}$, $\pi_{2}$, $\pi_{3}$, $\pi_{4}$) were considered, which are listed in Table 5. We evaluate the performance under the two‐subclass approach. For K=4, we have 7 models with two subclasses of homogeneous RD within each subclass. With a model under the common RD assumption, we applied 8 models in total in the two‐subclass approach. Here, we selected the model with the minimum GIC among the 8 models. We also applied the all‐subclass approach, in which all possible subclassifications were considered as candidate models, and the best one was selected with respect to the GIC. We evaluated the proportion with which each model was selected by the GIC over 10,000 simulated replicates. We also evaluated %Reject, which was defined as the proportion that the lower bound of the two‐sided 95% confidence interval of the basket‐specific response rate was greater than $\pi_{k,0}$ based on the best model. More specifically, the confidence interval of the response rate of each basket was constructed by $\pi_{k,0}$ plus the upper and lower bounds of the 95% confidence interval of the RD with the MH‐RD estimate based on the best model (partitioning) with the GIC.

**TABLE 5:** Settings of the simulation study: GN *and* GA *imply the global null and alternative hypotheses, respectively*.

Scenario	($n_{1}$, $n_{2}$, $n_{3}$, $n_{4}$)	($\pi_{1}$, $\pi_{2}$, $\pi_{3}$, $\pi_{4}$),	Correct model
1GN	(20, 20, 10, 10)	(0.1, 0.1, 0.1, 0.1)	[1,2,3,4]
2GA	(20, 20, 10, 10)	(0.3, 0.3, 0.3, 0.3)	[1,2,3,4]
3	(20, 20, 10, 10)	(0.1, 0.1, 0.3, 0.3)	[1,2/3,4]
4	(20, 20, 10, 10)	(0.1, 0.1, 0.1, 0.5)	[1,2,3/4]
5	(20, 20, 10, 10)	(0.1, 0.5, 0.5, 0.5)	[1/2,3,4]
6	(20, 20, 10, 10)	(0.1, 0.3, 0.3, 0.5)	[1/2,3/4]
7	(20, 20, 10, 10)	(0.1, 0.3, 0.5, 0.7)	[1/2/3/4]

### 5.2 Identification of the True Model

In Table 6, the frequencies of the selected models with the $GIC_{1}$ and $GIC_{2}$ under the all‐subclass and two‐subclass approaches over 10,000 simulated datasets are summarized. The $GIC_{2}$ under the all‐subclass approach selected the model [1/2/3/4] most frequently, as expected by the pathological nature of $GIC_{2}$. In theory, the model [1/2/3/4] should have the minimum $GIC_{2}$ asymptotically and then be selected. Since the bias term was estimated, there were some exceptions. We observed that the models other than [1/2/3/4] attained the minimum $GIC_{2}$ as seen in Table 6. On the other hand, the $GIC_{1}$ under the all‐subclass approach selected the correct model most frequently, except for scenarios 6 and 7. In scenario 6, the true model [1/2,3/4] was correctly selected with the $GIC_{1}$ only for 1389 simulated datasets. Consider the models [1/2,3,4] and [1,2,3/4], which were close to the true model [1/2,3/4]. They were selected for 3115 and 1243 datasets, respectively, and the former was selected the most frequently. The basket 4 was combined with the baskets 2 and 3 more frequently than the basket 1. Recall that $n_{1}=20$ and $n_{4}=10$. That is, baskets with a small number of subjects were more likely to be combined. It might not be a drawback in basket trials since it is desirable to borrow information for small baskets.

**TABLE 6:** Frequencies of the selected models as the best with respect to GIC in the simulation study; the true partitioning is highlighted in bold.

Scenario		All‐subclass				Two‐subclass			
		${GIC}_{1}$		${GIC}_{2}$		${GIC}_{1}$		${GIC}_{2}$	
True	rank	Frequency	Model	Frequency	Model	Frequency	Model	Frequency	Model
1GN	1	2477	**[1,2,3,4]**	2465	[1/2/3/4]	2452	**[1,2,3,4]**	1793	[1,2,4/3]
	2	1706	[1,2,3/4]	1482	[1/2/3,4]	1860	[1,2,4/3]	1787	[1,2,3/4]
**[1,2,3,4]**	3	1637	[1,2,4/3]	991	[1,2/3/4]	1802	[1,2,3/4]	1391	[1/2,3,4]
	4	1030	[1,2/3,4]	762	[1,3/2/4]	1155	[1,2/3,4]	1368	[1,3,4/2]
	5	624	[1,3,4/2]	759	[1,4/2/3]	733	[1/2,3,4]	1290	[1,2/3,4]
2GA	1	4770	**[1,2,3,4]**	4616	[1/2/3/4]	4628	**[1,2,3,4]**	1838	[1,2,3/4]
	2	871	[1,2,4/3]	1178	[1/2/3,4]	1007	[1,2,3/4]	1741	[1,2,4/3]
**[1,2,3,4]**	3	838	[1,2,3/4]	850	[1,2/3/4]	967	[1,2,4/3]	1408	[1,3,4/2]
	4	704	[1,2/3,4]	658	[1/2,3/4]	707	[1,2/3,4]	1368	[1/2,3,4]
	5	655	[1/2,3,4]	619	[1,4/2/3]	698	[1,3,4/2]	1247	[1,4/2,3]
3	1	3134	**[1,2/3,4]**	4912	[1/2/3/4]	3655	**[1,2/3,4]**	3886	**[1,2/3,4]**
	2	1366	[1,2,3,4]	1471	[1,2/3/4]	1380	[1,2,3,4]	1619	[1,2,3/4]
**[1,2/3,4]**	3	949	[1,2,4/3]	1344	[1/2/3,4]	1148	[1,2,3/4]	1604	[1,2,4/3]
	4	943	[1,2,3/4]	413	[1/2,3/4]	1144	[1,3,4/2]	1165	[1,3,4/2]
	5	859	[1/2/3,4]	408	[1,3/2/4]	1139	[1,2,4/3]	1124	[1/2,3,4]
4	1	3506	**[1,2,3/4]**	5014	[1/2/3/4]	6385	**[1,2,3/4]**	7463	**[1,2,3/4]**
	2	2254	[1,2/3/4]	1712	[1,2/3/4]	749	[1,2/3,4]	699	[1,2/3,4]
**[1,2,3/4]**	3	924	[1,3/2/4]	1348	[1,3/2/4]	656	[1,2,4/3]	457	[1,3/2,4]
	4	891	[1/2,3/4]	1253	[1/2,3/4]	476	[1,3/2,4]	422	[1,4/2,3]
	5	529	[1,2/3,4]	363	**[1,2,3/4]**	448	[1,4/2,3]	350	[1/2,3,4]
5	1	5999	**[1/2,3,4]**	6622	[1/2/3/4]	7674	**[1/2,3,4]**	7930	**[1/2,3,4]**
	2	751	[1/2,3/4]	1509	[1/2/3,4]	737	[1,3/2,4]	654	[1,3/2,4]
**[1/2,3,4]**	3	746	[1/2,4/3]	823	[1/2,3/4]	726	[1,4/2,3]	646	[1,4/2,3]
	4	670	[1/2/3,4]	659	[1/2,4/3]	340	[1,2/3,4]	295	[1,2/3,4]
	5	640	[1,4/2,3]	241	**[1/2,3,4]**	185	[1,3,4/2]	217	[1,3,4/2]
6	1	3115	[1/2,3,4]	6665	[1/2/3/4]	4298	[1/2,3,4]	4598	[1/2,3,4]
	2	1389	**[1/2,3/4]**	999	[1/2/3,4]	1899	[1,2,3/4]	2143	[1,2,3/4]
**[1/2,3/4]**	3	1243	[1,2,3/4]	916	**[1/2,3/4]**	1576	[1,3/2,4]	1584	[1,3/2,4]
	4	1242	[1,3/2,4]	452	[1,3/2/4]	1283	[1,2/3,4]	1182	[1,2/3,4]
	5	1077	[1,2/3,4]	343	[1,2/3/4]	535	[1,2,3,4]	209	[1,2,4/3]
7	1	2648	[1/2,3/4]	7682	**[1/2/3/4]**	4146	[1,2/3,4]	3864	[1/2,3,4]
	2	2074	[1,2/3,4]	1143	[1/2/3,4]	3593	[1/2,3,4]	3693	[1,2/3,4]
**[1/2/3/4]**	3	2034	[1/2/3,4]	599	[1/2,3/4]	1940	[1,2,3/4]	2186	[1,2,3/4]
	4	1320	[1/2,3,4]	364	[1,2/3/4]	196	[1,3/2,4]	159	[1,3/2,4]
	5	662	[1,2,3/4]	62	[1,2/3,4]	69	[1,2,4/3]	81	[1,2,4/3]

With the $GIC_{2}$ under the two‐subclass approach, incorrect models were selected most frequently even when the true model was included in the candidate models of the two‐subclass approach. For example, in the scenario 1GN with the true model [1,2,3,4], the models [1,2,4/3] and [1,2,3/4] were selected frequently. The selected model had an isolated basket and thus with the pathological nature of the $GIC_{2}$, the penalty term might not work well and then the $GIC_{2}$ failed to select the correct model. Similar observation was obtained for the scenario 2GA. In the scenarios 3,4, and 5, the true model was expressed as a model in the candidates under the two‐subclass approach and the $GIC_{2}$ successfully selected the true model most frequently.

### 5.3 Identification of Effective Baskets

In Table 7, we summarized %Reject of our proposed methods. We attach the results with the Bayesian methods in Table 2 of Zhou and Ji [13] for reference; SeparateBeB, in which conjugate beta‐prior distributions were updated separately by baskets, Bayesian Hierarchical Model (BHM) [12], EXNEX [9], BLAST [6] and RoBoT [13]. See Section 3.1 of Zhou and Ji [13] for the settings of parameters in each model. Pohl et al. [18] classified the existing Bayesian methods into the three groups; the first was the Bayesian hierarchical model with the normal distribution for the transformed response rate, the second was modeled to the response rate directly and the third was based on hypothesis‐driven sharing. BHM, EXNEX and BLAST were in the first group and RoBoT was in the third one. Here, we also applied the MEM [7]. It allows that baskets are a mixture of multiple sources of exchangeable subclasses and was classified into the second class of Pohl et al. [18]. We note that the definition of %Reject is different between the frequentist methods and the Bayesian methods. For our method, %Reject is defined as the proportion that the lower bound of the two‐sided 95% confidence interval for the basket‐specific response rate was greater than $\pi_{k,0}$ with the MH‐RD estimator based on the best model with respect to the GIC. For example, if we selected the model [1,2/3,4], the basket‐specific response rate for the basket k (k=1,2) was estimated by ${\hat{\Delta }}_{1,2}^{RD}+\pi_{k,0}$ and the confidence interval for $\pi_{k}$ was constructed accordingly with the confidence interval of $\Delta_{1,2}^{RD}$. On the other hand, for the Bayesian methods, Zhou and Ji [13] defined %Reject as the proportion of $P(\pi_{k}>\pi_{k,0}|data)>q$, where the probability was evaluated with the posterior distribution of each Bayesian method and q was determined so as to maintain the family‐wise error rate of 5% under the 1GN scenario. For BHM, EXNEX, BLAST and RoBot, the values for q are presented in Section 3 of Zhou and Ji (2021). For MEM, q=0.982. We summarize the results by the scenarios.

**TABLE 7:** Results of the simulation study for performance of basket‐specific inference; the first column of each scenario shows the four baskets and the basket surrounded by [ ] had a response rate $\pi_{k}$ greater than $\pi_{k,0}$ (see Table 5).

Scenario	Method	%Reject				100*bias			
1GN		1	2	3	4	1	2	3	4
	Separate BeB	1.4	1.2	1.2	1.3	0.08	−0.22	0.13	0.40
	BHM	2.7	2.7	2.2	2.3	−0.04	−0.03	0.10	0.17
	EXNEX	1.3	1.3	1.2	1.3	0.24	0.03	0.66	0.87
	BLAST	2.4	1.6	1.8	1.9	−0.05	−0.15	0.06	0.18
	RoBOT	1.5	1.3	1.0	1.3	−0.55	−0.79	−0.55	−0.29
	MEM	2.2	1.6	1.6	1.8	0.80	0.46	1.04	1.14
	GIC1 (all subclass)	2.0	1.8	1.7	1.6	0.59	0.49	−1.08	−1.02
	GIC2 (all subclass)	0.6	0.5	0.3	0.3	0.07	−0.02	−0.02	−0.02
	GIC1 (two subclass)	1.6	1.5	1.6	1.6	0.60	0.62	−1.33	−1.18
	GIC2 (two subclass)	1.7	1.6	1.7	1.6	0.10	0.09	−0.29	−0.16
2GN		[1]	[2]	[3]	[4]	[1]	[2]	[3]	[4]
	Separate BeB	56.7	54.4	33.7	34.0	0.07	−0.38	0.03	0.48
	BHM	91.8	92.0	85.4	86.2	0.03	−0.10	−0.10	−0.11
	EXNEX	60.8	58.6	34.7	36.0	−0.31	−0.69	−0.73	−0.39
	BLAST	90.0	90.1	80.9	80.9	0.04	−0.14	−0.08	0.14
	RoBOT	72.8	69.5	45.4	45.6	0.48	0.04	1.11	1.55
	MEM	90.2	87.8	81.6	81.4	0.57	−0.01	0.26	0.02
	GIC1 (all subclass)	80.6	80.4	76.7	77.0	0.02	−0.07	0.17	−0.01
	GIC2 (all subclass)	52.0	51.5	32.3	29.6	−0.08	−0.08	0.28	0.11
	GIC1 (two subclass)	81.5	81.2	77.2	76.8	0.10	0.11	−0.09	−0.13
	GIC2 (two subclass)	73.0	72.0	63.6	62.7	0.16	0.02	−0.04	−0.12
3		1	2	[3]	[4]	1	2	[3]	[4]
	Separate BeB	1.4	1.4	33.2	34.1	0.09	−0.20	0.02	0.45
	BHM	12.7	11.2	41.6	40.8	3.83	3.85	−7.22	−7.57
	EXNEX	1.4	1.5	33.4	34.3	0.85	0.63	−1.96	−1.61
	BLAST	9.3	8.3	40.0	40.1	3.08	3.02	−6.18	−6.01
	RoBOT	3.0	2.1	34.8	36.1	0.84	0.56	0.10	0.54
	MEM	8.5	8.0	38.6	38.6	3.41	3.03	−4.69	−4.78
	GIC1 (all subclass)	5.8	5.9	41.8	42.0	0.79	0.85	−1.73	−1.73
	GIC2 (all subclass)	0.8	0.6	20.0	19.7	−0.14	−0.06	0.09	0.13
	GIC1 (two subclass)	7.9	7.8	41.6	41.5	1.31	1.26	−2.85	−2.76
	GIC2 (two subclass)	6.5	6.3	40.8	41.0	0.90	0.80	−2.00	−1.86
4		1	2	3	[4]	1	2	3	[4]
	Separate BeB	1.4	1.3	1.2	82.7	0.08	−0.21	0.12	0.60
	BHM	7.9	6.5	6.0	67.3	2.46	2.40	3.27	−12.67
	EXNEX	1.4	1.4	1.1	81.0	0.56	0.32	1.21	−2.96
	BLAST	6.3	4.7	4.3	73.3	1.91	1.79	2.73	−9.84
	RoBOT	2.1	2.0	1.3	81.1	0.40	0.13	0.62	0.42
	MEM	2.8	2.2	3.1	72.8	1.70	1.39	2.42	−5.46
	GIC1 (all subclass)	3.6	3.7	6.3	67.5	0.54	0.63	−0.35	−2.19
	GIC2 (all subclass)	0.5	0.5	0.6	62.2	−0.04	0.10	−0.04	−0.29
	GIC1 (two subclass)	7.0	7.0	9.2	67.4	0.80	0.85	1.65	−4.93
	GIC2 (two subclass)	5.4	5.4	7.3	68.7	0.25	0.30	1.53	−2.62
5		1	[2]	[3]	[4]	1	[2]	[3]	[4]
	Separate BeB	1.4	97.8	82.0	82.3	0.07	−0.51	−0.06	0.59
	BHM	18.3	99.4	92.4	93.6	9.01	−4.44	−5.06	−4.67
	EXNEX	1.8	98.0	83.7	84.0	1.23	−1.55	−2.12	−1.52
	BLAST	16.2	99.3	92.5	93.8	6.87	−3.27	−4.12	−3.54
	RoBOT	5.3	98.9	85.6	86.4	1.89	−0.50	0.16	0.79
	MEM	9.0	98.6	92.1	91.5	3.99	−1.14	−2.02	−1.70
	GIC1 (all subclass)	4.4	97.7	90.2	90.1	0.71	−0.09	−0.79	−0.71
	GIC2 (all subclass)	0.6	95.2	69.5	68.2	−0.05	−0.10	0.12	−0.08
	GIC1 (two subclass)	6.4	97.8	91.7	92.0	1.19	0.34	−1.46	−1.29
	GIC2 (two subclass)	5.4	98.0	92.4	92.5	0.96	0.43	−1.29	−1.18
6		1	[2]	[3]	[4]	1	[2]	[3]	[4]
	Separate BeB	1.3	54.6	33.0	82.1	0.06	−0.37	0.03	0.60
	BHM	23.3	79.4	65.4	92.1	8.18	−1.71	−1.65	−11.45
	EXNEX	1.9	56.7	34.6	83.5	1.33	−0.77	−0.75	−3.12
	BLAST	18.2	76.8	61.4	91.3	6.32	−1.29	−1.29	−9.04
	RoBOT	4.5	64.5	39.0	85.3	1.66	−0.08	0.81	0.71
	MEM	15.4	73.0	62.1	89.8	5.44	−0.37	−0.46	−7.30
	GIC1 (all subclass)	9.9	68.8	65.7	93.2	1.37	0.28	0.00	−4.02
	GIC2 (all subclass)	1.0	45.9	26.0	69.7	−0.13	−0.09	−0.07	−0.22
	GIC1 (two subclass)	13.4	72.9	68.0	93.8	2.42	1.06	0.49	−6.78
	GIC2 (two subclass)	10.2	72.5	66.6	92.6	2.05	1.23	0.47	−6.36
7		1	[2]	[3]	[4]	1	[2]	[3]	[4]
	Separate BeB	1.4	55.0	81.1	98.7	0.08	−0.36	−0.07	−0.48
	BHM	13.6	75.4	91.1	99.5	6.45	0.61	−4.71	−10.90
	EXNEX	1.5	56.6	83.6	98.9	1.18	−0.26	−2.34	−4.88
	BLAST	11.5	75.1	91.2	99.7	5.05	0.56	−3.63	−8.79
	RoBOT	5.0	64.9	85.8	99.0	1.84	−0.03	0.14	−0.44
	MEM	11.8	63.7	88.6	99.6	4.71	0.91	−3.49	−6.46
	GIC1 (all subclass)	8.9	58.6	92.8	99.7	1.54	0.38	−0.31	−2.75
	GIC2 (all subclass)	1.3	42.5	69.3	96.4	0.05	0.10	0.40	0.11
	GIC1 (two subclass)	19.9	57.7	91.4	99.7	4.75	0.18	−0.66	−9.21
	GIC2 (two subclass)	19.7	59.8	90.7	99.6	4.59	0.83	−1.71	−9.12

Scenario 1GN: %Reject was less than 2.5% for all our methods. Thus, our methods could control false identification of effective baskets when all the baskets were ineffective. The Bayesian methods also protected too optimistic identification.

Scenario 2 GA: In this scenario, all the baskets were consistently effective. BHM, BLAST and MEM performed similarly well and outperformed the others. The $GIC_{1}$ improved the performance of the $GIC_{2}$ under both the all‐subclass and the two‐subclass approaches. The $GIC_{1}$ successfully borrowed more information than the $GIC_{2}$.

Scenario 3: The $GIC_{2}$ under the all‐subclass approach gave too conservative results. It was impacted by the pathological nature of the $GIC_{2}$; as presented in Table 6, the model [1/2/3/4] was most frequently selected. The $GIC_{1}$ successfully borrowed information and the $GIC_{1}$ under all‐subclass approach seemed to perform better than BHM, BLAST and MEM; the $GIC_{1}$ had comparable %Rejects for the baskets 3 and 4 and smaller ones for the baskets 1 and 2. EXNEX and RoBOT had better and well‐controlled %Reject for the baskets 1 and 2, whereas that for the baskets 3 and 4 (power) was less than the $GIC_{1}$ under the all‐subclass approach.

Scenarios 4 and 5: In both scenarios, the true model had an isolated basket; the true models were [1,2,3/4] and [1/2,3,4] for the scenarios 4 and 5, respectively. Note that the 4th basket had $n_{4}=10$ subjects. In Scenario 4, under the all‐subclass approach, the $GIC_{2}$ had very conservative %Rejects for noneffective baskets (baskets 1,2 and 3) and seemed to be borrowing less information. The $GIC_{1}$ seemed to borrow more information. However, improvement of %Reject for the basket 4 was not substantial. On the other hand, in scenario 5, there was a substantial improvement in %Reject for basket 4. In scenario 5, basket 4 was not isolated, and then more information was borrowed with the $GIC_{1}$. In these scenarios, our methods did not uniformly dominate the other Bayesian methods. We observed that our methods outperformed BHM and MEM.

Scenarios 6 and 7: In these scenarios, there were an isolated basket and subclasses of more than 2. The proposed method under the two‐subclass approach did not work well; the %Reject for noneffective baskets could inflate the nominal level of 2.5% substantially with $GIC_{1}$, as well as $GIC_{2}$. With the all‐subclass approach, inflation of the type 1 error rate for basket 1 was observed for $GIC_{1}$, but the amount of inflation was smaller than with the two‐subclass approach. Since $GIC_{2}$ likely selects a model of isolated baskets by its pathological nature, $GIC_{2}$ with the all‐subclass approach maintained a nominal type 1 error rate for basket 1. On the other hand, it had less powers, borrowing little information across baskets. Our proposed methods did not uniformly dominate the Bayesian methods. The $GIC_{1}$ under the all‐subclass approach outperformed BHM, BLAST, and MEM and was comparable to the other Bayesian methods.

In summary, the $GIC_{1}$ successfully borrowed information across studies, whereas the $GIC_{2}$ less effectively. The two‐subclass approach performs comparable or may outperform the all‐subclass approach when the baskets have two subclasses of homogeneous baskets. On the other hand, when they consist of more than two homogeneous subclasses, the two‐subclass approach may have serious false‐positive findings for noneffective baskets. Then, we recommend using the $GIC_{1}$ with the all‐subclass approach in general. No methods including the Bayesian methods did not dominate the others uniformly and the performance was strongly dependent on the situations.

## 6 Discussion

In this paper, we proposed an alternative information criterion $GIC_{1}$ to solve an issue in the $GIC_{2}$ by Hattori and Morita [23]. Our numerical studies revealed that the new information criterion $GIC_{1}$ successfully borrowed more information across baskets and outperformed the $GIC_{2}$, particularly with the all‐subclass approach, in clustering baskets and then identifying baskets effective to the treatment. Hattori and Morita [23] proposed to select the best model over all the models of two subclasses and the one under the common parameter assumption (the two‐subclass approach), considering classification into two subclasses of effective and ineffective baskets. It worked rather well since this approach excluded the model consisting of only isolated baskets such as [1/2/3/4], which inappropriately minimize the $GIC_{2}$ without a relevant penalty for model complexity (a pathological nature of $GIC_{2}$). However, the candidate models for the two‐subclass approach also include models of isolated baskets, and then the pathological nature of $GIC_{2}$ may have certain impacts. In addition, the class of the models under the two‐subclass approach is restrictive, and one may fail to identify the true structure when there are more than two subclasses of baskets. The $GIC_{1}$ overcomes these difficulties. It enables us to search for an appropriate model in a more flexible manner without restricting the class of candidate models.

As outlined in Section 2, the one‐sample MH procedure consists of three steps. The steps (i) and (ii) are testing the global hypothesis and estimating the overall treatment effect. This part is a natural extension of the standard analysis of the single‐arm trial with the exact binomial testing. The step (iii) is to identify the effective baskets and is an exploratory part of the procedure after the steps (i) and (ii). The procedure looks like the standard confirmatory trials. For example, consider a confirmatory Phase 3 trial with a time‐to‐event endpoint. The study is usually designed so that the testing hypothesis is valid; the log‐rank test is often used to test the null hypothesis of no treatment effect. To summarize the overall treatment effect, one may often need to make some extra assumptions. The hazard ratio is often used to efficiently summarize the overall treatment effect, assuming proportional hazards at the design stage. However, the summary of the treatment effect with the hazard ratio may not be appealing; the proportional hazards assumption may not hold, and the treatment effect may be limited to some subgroups. For the former issue, some efforts have been taken to preserve the interpretability of the misspecified hazard ratio estimate [32, 33, 34]. For example, Hattori and Henmi [33] proposed to estimate the hazard ratio with the weighted score equation for the partial likelihood and showed that it consistently estimates the hazard ratio if the proportional hazards assumption holds and converges in probability to a geometric mean of time‐specific hazard ratio over time even if the proportional hazards assumption is violated. For the second issue, subgroup analysis is often conducted as a post‐hoc analysis of an exploratory nature. The one‐sample MH procedure follows this strategy; one must rely on the common RD or RR assumption and employ the MH‐RD or MH‐RR estimator, which preserves the interpretability as the weighted average of the basket‐specific RD or RR over baskets even if the common parameter assumption is violated. Presenting basket‐specific estimates as in Tables 2 and 4 corresponds to subgroup analysis, inferring the common parameter. In this paper, we propose a tool supporting this consideration. Since the basket trials have more exploratory aspects than the confirmatory Phase 3 studies, this addition would be an important contribution to the basket trial methodology. In step (iii), we used two‐sided 95% confidence intervals to present basket‐specific treatment effects as often done in subgroups analysis. Our investigation for the identification of effective baskets was limited to this simple strategy. Indeed, we observed that our method suffered from false‐positive findings in our simulation study. Bayesian methods also could not control the false‐positive findings in basket‐wise inference, and then it was difficult to compare the performance of the Bayesian methods and our proposed method in a rigorous manner. Overall, the effective basket identification with the naive two‐sided 95% confidence intervals of our proposed method worked comparably with the Bayesian methods. It would be valuable to put more efforts to realize effective basket identification methods for better control of false‐positive findings and make more formal comparisons.

As the class of candidate models (partitionings), we considered the two‐subclass and the all‐subclass approaches. In basket trials, one may hope to discriminate baskets into effective and ineffective ones. In this sense, the two‐subclass approach might be more appealing. Indeed, the all‐subclass approach might lead to a more precise partitioning than the two‐subclass approach. It might lead to less precise estimates of basket‐specific response rate. On the other hand, the two‐subclass approach might fail to identify the true structure of the partitioning and be subject to severe false‐positive detection of effective baskets. Thus, in general, we recommend applying the $GIC_{1}$ under the all‐subclass approach. A practical approach is to list several top models with respect to the $GIC_{1}$ as done in the Example section, and does not stick to only the best model. In the first example of Vemurafenib, we observed that the model [1,2,6/3,4,5], which was the best model by the two‐subclass approach with $GIC_{2}$, had a close $GIC_{1}$ value to the best model [1,2,6/3,4/5]. In this case, the effectiveness of [1, 2, 6] was supported by both approaches, and basket 5 is likely to be ineffective since its pooling to the ineffective baskets [3, 4] did not change the GIC value so much. Such consideration would be useful to draw a robust decision.

A disadvantage of our method is that it does not have the ability to flexibly incorporate prior information, whereas Bayesian methods would be able to do so in principle. Thus, if there are some relevant prior knowledge on efficacy of baskets, Bayesian approach would bring us more benefits. On the other hand, single‐arm basket trials in oncology are often conducted in early Phase 2. Usually, only limited results of Phase 1 studies are available. Then, it is not necessarily easy to incorporate prior knowledge even in Bayesian frameworks. If this is the case, the one‐sample MH procedure might be beneficial with its simplicity, comparable performance with Bayesian methods and confirmatory nature. It is very important to clarify how to incorporate prior information into the one‐sample MH procedure. An idea is to define a relevant class of candidate models alternative to the all‐subclass and the two‐subclass approaches. If there is a strong brief that the treatment should act similarly for some baskets, say baskets 1 and 2, one may exclude models separating the baskets 1 and 2. Or, if enrollment of patients in some basket is suggested difficult and limited at the design‐stage, one may avoid any models with the basket as isolated one. Such extensions should be addressed in future research.

## Funding

This work was supported by the Grant‐in‐Aid for Scientific Research, the Ministry of Education, Science, Sports and Technology of Japan (Grant No. 25K03086).

## Conflicts of Interest

The authors declare no conflicts of interest.

## Data Availability

We used a dataset available to the public. A R code to implement the proposed method is available at https://github.com/tanamikan/BasketMHGIC.

## Appendix A A Lemma

We present a key lemma given by Konishi and Kitagawa [29] to derive the GIC for M‐estimators. Here we consider a general setting and notations independent of the main body of the present paper. Let N be the number of samples and $Z_{i},i=1,2,\ldots ,N$ be the independently and identically samples from a population. Let g(z) and G(z) be the probability density and the cumulative distribution functions of $Z_{i}$, respectively. We assume a parametric model {f(z;θ)}, where f(z;θ) is a probability density function and θ is a p‐dimensional unknown parameter. We assume that the parameter θ is estimated by solving the following estimating equation;

(A1)
$$\begin{array}{l} \overset{N}{\underset{i=1}{\sum }}\psi (Z_{i};\theta )=0, \end{array}$$

where ψ(z;θ) is a p‐dimensional function. Let the solution to (A1) is denoted by $\hat{\theta }$. We prepare a lemma. See Theorem 3.1 of Konishi and Kitagawa [29]. Lemma:

The GIC is given by

(A2)
$$\begin{array}{l} GIC=-\overset{N}{\underset{i=1}{\sum }}\log f(Z_{i};\theta )+bias, \end{array}$$

with $bias=tr\left[{\hat{M}}^{-1}\hat{L}\right]$, where

$$\begin{array}{l} \hat{M}=-\frac{1}{n}\overset{N}{\underset{i=1}{\sum }}\frac{\partial \psi^{T}(Z_{i};\theta )}{\partial \theta }{|}_{\theta =\hat{\theta }} \end{array}$$

and

$$\begin{array}{l} \hat{L}=n^{-1}\overset{n}{\underset{i=1}{\sum }}\psi (Z_{i};\theta )\frac{\partial }{\partial \theta^{T}}\log f(Z_{i};\theta ){|}_{\theta =\hat{\theta }} \end{array}$$

## Appendix B Derivation of the $GIC_{2}$

The derivation of the $GIC_{2}$ was outlined in Appendix D of Hattori and Morita [23]. By setting N=K and $Z_{k}=y_{k}$ for k=1,2,..,K, the estimating equation (6) is represented in the form of (A1) as $\sum_{k=1}^{K}\psi (y_{k};\Delta )=\sum_{k=1}^{K}\{R_{k}-\Delta S_{k}\}=0$. Applying the lemma with $\psi (y_{k};\Delta )=R_{k}-\Delta S_{k}$ in (6), the $GIC_{2}$ was derived.

Since the lemma (Theorem 3.1 of Konishi and Kitagawa [29]) is described for the independently and identically distributed samples, a careful application is needed for the $GIC_{2}$. In this appendix, we present a more formal derivation, which was not implicitly presented by Hattori and Morita [23]. Recall that $Y_{k}$ is assumed to follow the binomial distribution $Bin(n_{k},\pi_{k})$ conditional on $n_{k}$. Let the probability mass function of $Bin(n_{k},\pi_{k})$ denoted by $f(y_{k}|n_{k};\pi_{k})=f(y_{k}|n_{k};h_{k}(\Delta ))$. Regarding $\left\{(y_{k},n_{k})\right\}$ as a realization from a population, let the probability mass function of the joint distribution of $\left(y_{k},n_{k}\right)$ denoted by $f(y_{k},n_{k};\Delta )$ and the marginal mass function of $n_{k}$ denoted by $f(n_{k})$, which is assumed free from Δ.

Then, applying the lemma to the independently and identically distributed sample $\left\{(y_{k},n_{k})\right\}$, the first term of (A2) is given by

(B1)
$$\begin{array}{l} -\;\overset{K}{\underset{k=1}{\sum }}\log f(y_{k},n_{k};\Delta )=-\overset{K}{\underset{k=1}{\sum }}\log f(y_{k}|n_{k};h_{k}(\Delta ))-\overset{K}{\underset{k=1}{\sum }}\log f(n_{k}). \end{array}$$

The first term of the right‐hand side of (B1) agrees with the first term of (7). Note that the second term does not depend on the model and then can be ignored in comparing models.

By simple algebra, $\hat{M}=-K^{-1}\sum_{k=1}^{K}S_{k}$ and

$$\begin{array}{ll} \hat{L} & =K^{-1}\overset{K}{\underset{k=1}{\sum }}\psi (y_{k};\Delta )\frac{\partial }{\partial \Delta }\{\log p(y_{k}|n_{k};h_{k}(\Delta ))-\log p(n_{k})\}{|}_{\Delta =\hat{\Delta }}\\ & =K^{-1}\overset{K}{\underset{k=1}{\sum }}\psi (y_{k};\Delta )\frac{\partial }{\partial \Delta }\log p(y_{k}|n_{k};h_{k}(\Delta )){|}_{\Delta =\hat{\Delta }} \end{array}$$

holds. Then the $GIC_{2}$ (7) is justified from the lemma in Appendix A.

## Appendix C Derivation of the $GIC_{1}$

Set $Z_{i}=(D_{1}^{\left(i\right)},D_{2}^{\left(i\right)},\ldots ,D_{K}^{\left(i\right)},Y^{\left(i\right)})$ and $\psi (Z_{i};\Delta )=\sum_{k=1}^{K}w_{k}D_{k}^{\left(i\right)}\{Y^{\left(i\right)}-h_{k}(\Delta )\}$ in (13). Then, the estimating equation in (13) is represented as (A1). The log‐likelihood function is given by

$$\begin{array}{ll} & \overset{K}{\underset{k=1}{\sum }}\log\frac{n_{k}}{Y_{k}(n_{k}-Y_{k})}+\overset{n}{\underset{i=1}{\sum }}\overset{K}{\underset{k=1}{\sum }}D_{k}^{\left(i\right)}\{Y^{\left(i\right)}\log h_{k}(\Delta )\\ & \;+(1-Y^{\left(i\right)})\log\{1-h_{k}(\Delta )\}\}. \end{array}$$

Then, by simple algebra,

$$\begin{array}{ll} \hat{M} & =-\frac{1}{n}\overset{n}{\underset{i=1}{\sum }}\overset{K}{\underset{k=1}{\sum }}w_{k}D_{k}^{\left(i\right)}{\dot{h}}_{k}(\hat{\Delta }),\\ & =\frac{1}{n}\overset{n}{\underset{i=1}{\sum }}\overset{K}{\underset{k=1}{\sum }}D_{k}^{\left(i\right)}=\frac{1}{n}\overset{K}{\underset{k=1}{\sum }}n_{k}=\frac{1}{n}\overset{K}{\underset{k=1}{\sum }}S_{k}, \end{array}$$

where ${\dot{h}}_{k}(x)=dh_{k}(x)/dx$ and $w_{k}{\dot{h}}_{k}(\Delta )=1$ holds both for the MH‐RD and the MH‐iwRR noting that $w_{k}=1$ and ${\dot{h}}_{k}(x)=1$ for the MH‐RD, and $w_{k}=\pi_{k,0}^{-1}$ and ${\dot{h}}_{k}(x)=\pi_{k,0}$ for the MH‐iwRR. Furthermore, noting again that $w_{k}{\dot{h}}_{k}(\Delta )=1$ both for the MH‐RD and the MH‐iwRR estimators, it holds that

$$\begin{array}{ll} \hat{L} & =\frac{1}{n}\overset{n}{\underset{i=1}{\sum }}\left[\overset{K}{\underset{k=}{\sum }}w_{k}D_{k}^{\left(i\right)}\{Y^{\left(i\right)}-h_{k}(\Delta )\}\right.\times \left.\overset{K}{\underset{k=1}{\sum }}{\dot{h}}_{k}(\hat{\Delta })D_{k}^{\left(i\right)}\frac{Y^{\left(i\right)}-h_{k}(\hat{\Delta })}{h_{k}(\hat{\Delta })\{1-h_{k}(\hat{\Delta })\}}\right]\\ & =\frac{1}{n}\overset{n}{\underset{i=1}{\sum }}\overset{K}{\underset{k=1}{\sum }}D_{k}^{\left(i\right)}\frac{{\left\{Y^{\left(i\right)}-h_{k}(\Delta )\right\}}^{2}}{h_{k}(\Delta )\{1-h_{k}(\Delta )\}}\\ & =\frac{1}{n}\overset{K}{\underset{k=1}{\sum }}\left[\frac{\{1-2h_{k}(\Delta )\}Y_{k}+h_{k}{\left(\Delta \right)}^{2}n_{k}}{h_{k}(\Delta )\{1-h_{k}(\Delta )\}}\right]. \end{array}$$

It completes the derivation of (14).
